# Glycine methyl ester hydro­chloride

**DOI:** 10.1107/S1600536809028414

**Published:** 2009-07-25

**Authors:** Sérgio M. F. Vilela, Filipe A. Almeida Paz, João P. C. Tomé, Verónica de Zea Bermudez, José A. S. Cavaleiro, João Rocha

**Affiliations:** a Department of Chemistry, University of Aveiro, CICECO, 3810-193 Aveiro, Portugal; b Department of Chemistry, University of Aveiro, QOPNA, 3810-193 Aveiro, Portugal; c Department of Chemistry, CQ-VR, University of Trás-os-Montes e Alto Douro, 5001-801 Vila Real, Portugal

## Abstract

The title compound [systematic name: (methoxy­carbonyl­meth­yl)ammonium chloride], crystallizes as a salt, C_3_H_8_NO_2_^+^·Cl^−^, with the charged species inter­acting mutually *via* strong and highly directional N^+^—H⋯Cl^−^ hydrogen bonds which lead to the formation of a supra­molecular tape running parallel to the *c* axis. Tapes close pack in the solid state mediated by multipoint recognition synthons based on weak C—H⋯O inter­actions and van der Waals contacts between adjacent methyl groups.

## Related literature

For related structures, see: Handelsman-Benory *et al.* (1995 ▶). For detailed background to the role of hydrogen bonds in the supra­molecular organization of organic crystals, see: Nangia & Desiraju (1998 ▶). For general background studies on crystal engineering approaches from our research group, see: Shi *et al.* (2008 ▶); Paz & Klinowski (2003 ▶); Paz *et al.* (2002 ▶). For a description of the Cambridge Structural Database, see: Allen (2002 ▶).
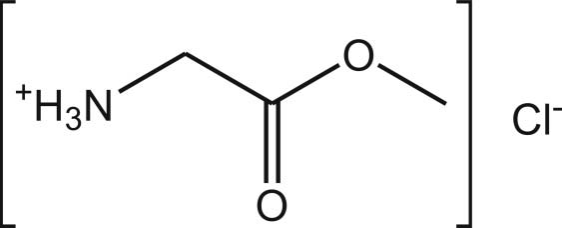


## Experimental

### 

#### Crystal data

C_3_H_8_NO_2_^+^·Cl^−^
*M* *_r_* = 125.55
Monoclinic, 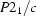

*a* = 8.352 (2) Å
*b* = 12.505 (3) Å
*c* = 5.6369 (14) Å
β = 99.730 (9)°
*V* = 580.3 (2) Å^3^
*Z* = 4
Mo *K*α radiation
μ = 0.55 mm^−1^
*T* = 150 K
0.08 × 0.02 × 0.02 mm

#### Data collection

Bruker X8 Kappa CCD APEXII diffractometer
Absorption correction: multi-scan (*SADABS*; Sheldrick, 1997 ▶) *T*_min_ = 0.957, *T*_max_ = 0.989
5189 measured reflections
1539 independent reflections
923 reflections with *I* > 2σ(*I*)
*R*_int_ = 0.070

#### Refinement

*R*[*F* ^2^ > 2σ(*F* ^2^)] = 0.069
*wR*(*F* ^2^) = 0.184
*S* = 1.08
1539 reflections
66 parameters
H-atom parameters constrained
Δρ_max_ = 1.05 e Å^−3^
Δρ_min_ = −0.55 e Å^−3^

### 

Data collection: *APEX2* (Bruker, 2006 ▶); cell refinement: *SAINT-Plus* (Bruker, 2005 ▶); data reduction: *SAINT-Plus*; program(s) used to solve structure: *SHELXTL* (Sheldrick, 2008 ▶); program(s) used to refine structure: *SHELXTL*; molecular graphics: *DIAMOND* (Brandenburg, 2009 ▶); software used to prepare material for publication: *SHELXTL*.

## Supplementary Material

Crystal structure: contains datablocks global, I. DOI: 10.1107/S1600536809028414/hg2539sup1.cif

Structure factors: contains datablocks I. DOI: 10.1107/S1600536809028414/hg2539Isup2.hkl

Additional supplementary materials:  crystallographic information; 3D view; checkCIF report

## Figures and Tables

**Table 1:** Hydrogen-bond geometry (Å, °)

*D*—H⋯*A*	*D*—H	H⋯*A*	*D*⋯*A*	*D*—H⋯*A*
N1—H1*A*⋯Cl1^i^	0.91	2.52	3.269 (4)	140
N1—H1*B*⋯Cl1^ii^	0.91	2.25	3.112 (4)	158
N1—H1*C*⋯Cl1	0.91	2.26	3.147 (4)	165
C1—H1*D*⋯Cl1^iii^	0.99	2.69	3.448 (4)	133
C1—H1*E*⋯O2^i^	0.99	2.51	2.928 (4)	105

Symmetry codes: (i) 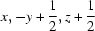; (ii) 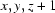; (iii) 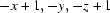.

**Fig. 1.**
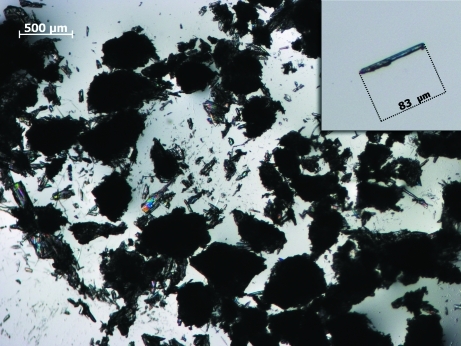
Optical photographs of the (micro-)crystalline material composing the title compound. The inset shows the single-crystal used for data collection, emphasizing morphology (thin needle) and small crystal size. Optical photographs were taken on a Stemi 2000 stereomicroscope equipped with Carl Zeiss lenses and a digital high-resolution AxioCam MRc5 digital camera connected to a personal computer.

**Fig. 2.**
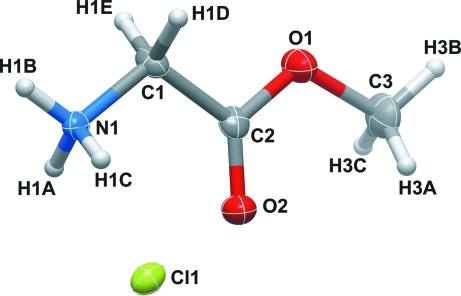
Asymmetric unit of the title compound. Non-hydrogen atoms are represented as thermal displacement ellipsoids drawn at the 50% probability level and hydrogen atoms as small spheres with arbitrary radii. The atomic labeling is provided for all atoms. Bond lengths and angles are provided in the following Tables.

**Fig. 3.**
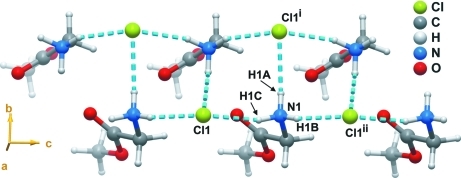
Schematic representation of the N+—H···Cl- hydrogen bonding interactions which lead to the formation of a one-dimensional supramolecular tape running parallel to the c axis. For details on the hydrogen bonding geometry see Table 1. Symmetry transformations used to generate equivalent atoms: (i) x, -y + 1/2, z+1/2; (ii) x, y, z+1.

**Fig. 4.**
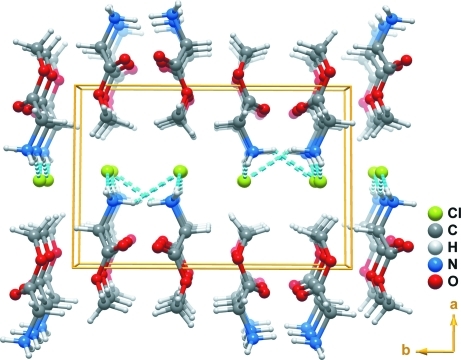
Perspective view along the [001] direction of the crystal packing of the title compound. N+—H···Cl- hydrogen bonds are represented as light-blue dashed lines.

## supplementary crystallographic information

### Comment

Following our interest in crystal engineering (Shi *et al.*, 2008; Paz & Klinowski, 2003; Paz *et al.*, 2002), we recently started to prepare novel organic ligands based on amino acid moieties, which can be ultimately employed as building units in the construction of multi-dimensional coordination polymers. Even though the latest release of the Cambridge Structural Database (CSD, Version of November 2008 with three updates; Allen, 2002) contains the remarkable number of 70 determinations of zwiterionic glycine, the structure of the title compound has never been reported to date. It is important to emphasize that crystallization of this compound is not trivial, typically leading to microcrystalline powders with needle-like crystal habit. Larger single crystals could only be obtained when a 1: 1 chloroform: methanol mixture was used (Fig. 1).

The asymmetric unit of the title compound, (I), is composed of a cationic glycinium methyl ester moiety and a chloride anion as depicted in Fig. 2. In the crystal structure, the three crystallographically independent N—H moieties are engaged in strong and highly directional [<(DHA) greater than 140°] N^+^—H···Cl^-^ hydrogen bonds with three symmetry-related Cl^-^ anions (Fig. 3). These interactions promote the formation of a tape of C_3_H_8_NO_2_^+.^Cl^-^ moieties running parallel to the *c* axis. It is noteworthy to emphasize that the N^+^—H···Cl^-^ interactions can be divided into two families according to the registered *d*_D···A_ distances: while the two outer interactions of the tape have interatomic distances ranging from 3.112 (4) to 3.112 (4) Å, the inner hydrogen bond is weaker with the corresponding value being 3.269 (4) Å. Indeed, FT—IR studies on the title compound support this assumption clearly revealing the presence of two stretching bands centered at *ca* 2628 and 2682 cm^-1^ and attributed to two ν(N^+^—H) families, respectively.

Hydrogen-bonded supramolecular C_3_H_8_NO_2_^+.^Cl^-^ chains close pack in the solid state mediated by a combination multipoint recognition synthons involving weak C—H···O hydrogen bonds (not shown; Nangia & Desiraju, 1998) and the need to effectively fill the available space (*i.e.*, van der Waals interactions; Fig. 4). This packing behavior is strikingly distinct from that reported by Handelsman-Benory *et al.* (1995): while in (I) all H atoms bound to nitrogen are engaged in N^+^—H···Cl^-^ interactions, in glycyl-glycyl-glycine methyl ester hydrochloride the N—H and C=O moieties from the middle of the chain establish further hydrogen bonding connections with neighboring molecules; the final connectivity of the compounds results in a three-dimensional network of strong and highly directional hydrogen bonds.

### Experimental

Glycine methyl ester hydrochloride (99% purity) was purchased from Sigma-Aldrich and used without further purification. Crystalline material suitable for single-crystal studies (Fig. 1) was isolated from the slow evaporation (at ambient temperature) of a 1: 1 chloroform: methanol solution.

Fourier-Transform Infrared (FT—IR) spectra were obtained as KBr (Aldrich 99%+, FT—IR grade) pellets using a Mattson 7000 instrument. Selected FT—IR bands (in cm^-1^): overtone ν(C=O) = 3400*br,m*; ν(N^+^—H) = 2682*m* and 2628*m*; ν(C=O) = 1749*vs*; ν*_asym_*(C—O—C) = 1259*vs*.

### Refinement

Hydrogen atoms bound to nitrogen and carbon were located at their idealized positions and were included in the final structural model in riding-motion approximation with C—H = 0.98 Å and N—H = 0.91 Å. The isotropic thermal displacement parameters for these atoms were fixed at 1.2 (for the —CH_2_— group) or 1.5 (for the pendant —NH_3_^+^ and —CH_3_ moieties) times *U*_eq_ of the atom to which they are attached.

### Crystal data

**Table:**

C_3_H_8_NO_2_^+^·Cl^−^	*F*(000) = 264
*M**_r_* = 125.55	*D*_x_ = 1.437 Mg m^−^^3^
Monoclinic, *P*2_1_/*c*	Mo *K*α radiation, λ = 0.71073 Å
Hall symbol: -P 2ybc	Cell parameters from 793 reflections
*a* = 8.352 (2) Å	θ = 3.0–24.8°
*b* = 12.505 (3) Å	µ = 0.55 mm^−^^1^
*c* = 5.6369 (14) Å	*T* = 150 K
β = 99.730 (9)°	Needle, colourless
*V* = 580.3 (2) Å^3^	0.08 × 0.02 × 0.02 mm
*Z* = 4	

### Data collection

**Table:**

Bruker X8 Kappa CCD APEXII diffractometer	1539 independent reflections
Radiation source: fine-focus sealed tube	923 reflections with *I* > 2σ(*I*)
graphite	*R*_int_ = 0.070
φ scans	θ_max_ = 29.1°, θ_min_ = 4.0°
Absorption correction: multi-scan (*SADABS*; Sheldrick, 1997)	*h* = −11→11
*T*_min_ = 0.957, *T*_max_ = 0.989	*k* = −17→16
5189 measured reflections	*l* = −7→7

### Refinement

**Table:**

Refinement on *F*^2^	Primary atom site location: structure-invariant direct methods
Least-squares matrix: full	Secondary atom site location: difference Fourier map
*R*[*F*^2^ > 2σ(*F*^2^)] = 0.069	Hydrogen site location: inferred from neighbouring sites
*wR*(*F*^2^) = 0.184	H-atom parameters constrained
*S* = 1.08	*w* = 1/[σ^2^(*F*_o_^2^) + (0.0649*P*)^2^ + 1.246*P*] where *P* = (*F*_o_^2^ + 2*F*_c_^2^)/3
1539 reflections	(Δ/σ)_max_ < 0.001
66 parameters	Δρ_max_ = 1.05 e Å^−^^3^
0 restraints	Δρ_min_ = −0.55 e Å^−^^3^

### Special details

**Table:**

Geometry. All e.s.d.'s (except the e.s.d. in the dihedral angle between two l.s. planes) are estimated using the full covariance matrix. The cell e.s.d.'s are taken into account individually in the estimation of e.s.d.'s in distances, angles and torsion angles; correlations between e.s.d.'s in cell parameters are only used when they are defined by crystal symmetry. An approximate (isotropic) treatment of cell e.s.d.'s is used for estimating e.s.d.'s involving l.s. planes.
Refinement. Refinement of *F*^2^ against ALL reflections. The weighted *R*-factor *wR* and goodness of fit *S* are based on *F*^2^, conventional *R*-factors *R* are based on *F*, with *F* set to zero for negative *F*^2^. The threshold expression of *F*^2^ > σ(*F*^2^) is used only for calculating *R*-factors(gt) *etc*. and is not relevant to the choice of reflections for refinement. *R*-factors based on *F*^2^ are statistically about twice as large as those based on *F*, and *R*- factors based on ALL data will be even larger.

### Fractional atomic coordinates and isotropic or equivalent isotropic displacement parameters (Å^2^)

**Table:**

	*x*	*y*	*z*	*U*_iso_*/*U*_eq_	
Cl1	0.47434 (13)	0.11638 (8)	0.24634 (19)	0.0210 (3)	
C1	0.7947 (5)	0.0942 (3)	0.8596 (8)	0.0200 (10)	
H1D	0.7834	0.0155	0.8502	0.024*	
H1E	0.8397	0.1135	1.0280	0.024*	
C2	0.9087 (5)	0.1308 (3)	0.6967 (8)	0.0190 (9)	
C3	1.1805 (6)	0.1209 (5)	0.6290 (10)	0.0348 (12)	
H3A	1.1348	0.1161	0.4573	0.052*	
H3B	1.2733	0.0723	0.6660	0.052*	
H3C	1.2163	0.1944	0.6682	0.052*	
N1	0.6341 (4)	0.1443 (3)	0.7897 (6)	0.0178 (8)	
H1A	0.6452	0.2167	0.7915	0.027*	
H1B	0.5683	0.1246	0.8954	0.027*	
H1C	0.5895	0.1225	0.6390	0.027*	
O1	1.0566 (4)	0.0911 (3)	0.7714 (6)	0.0295 (8)	
O2	0.8701 (4)	0.1866 (3)	0.5246 (6)	0.0251 (8)	

### Atomic displacement parameters (Å^2^)

**Table:**

	*U*^11^	*U*^22^	*U*^33^	*U*^12^	*U*^13^	*U*^23^
Cl1	0.0258 (6)	0.0195 (5)	0.0182 (6)	−0.0065 (5)	0.0049 (4)	−0.0014 (5)
C1	0.016 (2)	0.014 (2)	0.030 (3)	−0.0018 (17)	0.0031 (17)	0.0025 (17)
C2	0.021 (2)	0.0111 (19)	0.026 (3)	−0.0028 (16)	0.0058 (17)	−0.0028 (18)
C3	0.020 (2)	0.040 (3)	0.047 (3)	0.000 (2)	0.013 (2)	0.003 (3)
N1	0.0204 (19)	0.0166 (18)	0.017 (2)	0.0018 (14)	0.0050 (14)	−0.0001 (14)
O1	0.0223 (18)	0.0313 (18)	0.036 (2)	0.0042 (15)	0.0086 (14)	0.0102 (16)
O2	0.0268 (18)	0.0239 (17)	0.0253 (19)	0.0024 (14)	0.0065 (14)	0.0065 (14)

### Geometric parameters (Å, °)

**Table:**

C1—N1	1.472 (5)	C3—H3A	0.9800
C1—C2	1.502 (6)	C3—H3B	0.9800
C1—H1D	0.9900	C3—H3C	0.9800
C1—H1E	0.9900	N1—H1A	0.9100
C2—O2	1.194 (5)	N1—H1B	0.9100
C2—O1	1.332 (5)	N1—H1C	0.9100
C3—O1	1.461 (5)		
			
N1—C1—C2	110.5 (4)	H3A—C3—H3B	109.5
N1—C1—H1D	109.6	O1—C3—H3C	109.5
C2—C1—H1D	109.6	H3A—C3—H3C	109.5
N1—C1—H1E	109.6	H3B—C3—H3C	109.5
C2—C1—H1E	109.6	C1—N1—H1A	109.5
H1D—C1—H1E	108.1	C1—N1—H1B	109.5
O2—C2—O1	125.7 (4)	H1A—N1—H1B	109.5
O2—C2—C1	124.2 (4)	C1—N1—H1C	109.5
O1—C2—C1	110.1 (4)	H1A—N1—H1C	109.5
O1—C3—H3A	109.5	H1B—N1—H1C	109.5
O1—C3—H3B	109.5	C2—O1—C3	115.9 (4)
			
N1—C1—C2—O2	−5.0 (6)	O2—C2—O1—C3	0.9 (7)
N1—C1—C2—O1	175.7 (3)	C1—C2—O1—C3	−179.7 (4)

### Hydrogen-bond geometry (Å, °)

**Table:**

*D*—H···*A*	*D*—H	H···*A*	*D*···*A*	*D*—H···*A*
N1—H1A···Cl1^i^	0.91	2.52	3.269 (4)	140
N1—H1B···Cl1^ii^	0.91	2.25	3.112 (4)	158
N1—H1C···Cl1	0.91	2.26	3.147 (4)	165
C1—H1D···Cl1^iii^	0.99	2.69	3.448 (4)	133
C1—H1E···O2^i^	0.99	2.51	2.928 (4)	105

Symmetry codes: (i) *x*, −*y*+1/2, *z*+1/2; (ii) *x*, *y*, *z*+1; (iii) −*x*+1, −*y*, −*z*+1.
